# A Clinical Manual of Diseases of the Ear

**Published:** 1887-06

**Authors:** 


**A Clinical Manual of Diseases of the Ear.** By LAURENCE TRUMBULL, M. D., Ph. G., Aural Surgeon to the Jefferson Medical College Hospital, late Honorary President of the Otological Subsection of the British Medical Association at Cork, and Author of a Work on Hygiene of the Ear; with a colored lithographic plate and numerous illustrations on wood. Second edition. Philadelphia: J. B. Lippincott Company. 1887. Price, $3.00.

"The sale of as large an edition as seventeen hundred copies of the author's work shows the appreciation of the profession of its character and scope." This book, as its name implies, is more decidedly a clinical manual. The statements found in the work are based on facts obtained after careful research. Those who have attended Dr. Trumbull's clinical lectures at the Jefferson Medical College Hospital will certainly agree with the statement that the doctor is painstaking and careful in his methods of recording cases.

The various methods of treatment of the many ear diseases have been so accurately and fully described as to merit especia mention. An appendix with an elaborate description and numerous cuts of the methods of illuminating the ear, nose, throat, and eye, makes this valuable book doubly acceptable at this time. It is needless to state that it is a work which can be made of great service to the general practitioner.

